# Rare metastatic pancreatic tumors from lung cancer with cystic changes resembling intraductal papillary mucinous neoplasm: a case report

**DOI:** 10.1186/s40792-020-00870-5

**Published:** 2020-05-28

**Authors:** Akira Watanabe, Norifumi Harimoto, Takahiro Yamanaka, Norihiro Ishii, Mariko Tsukagoshi, Takamichi Igarashi, Norio Kubo, Kenichiro Araki, Aya Suzuki, Kenichiro Hara, Ken Shirabe

**Affiliations:** 1 Department of General Surgical Science, Division of Hepatobiliary and Pancreatic Surgery, Graduate School of Medicine, Gunma University, 3-39-15 Showamachi, Maebashi, Gunma 371-8511 Japan; 2 Department of General Surgical Science, Division of Hepatobiliary and Pancreatic Surgery, Graduate School of Medicine, Gunma University, 3-39-22 Showamachi, Maebashi, Gunma 371-8511 Japan; 3 Department of Innovative Cancer Immunotherapy, Gunma University, 3-39-15 Showamachi, Maebashi, Gunma 371-8511 Japan; 4 Department of Pathology, Gunma University Hospital, 3-39-15 Showamachi, Maebashi, Gunma 371-8511 Japan; 5 Division of Allergy and Respiratory Medicine, Integrative Center of Internal Medicine, Gunma University Hospital, 3-39-15 Showamachi, Maebashi, Gunma 371-8511 Japan

**Keywords:** Metastatic pancreatic tumor from lung cancer (MPTLC), Cystic tumor, Intraductal papillary mucinous neoplasm (IPMN), High-risk stigmata

## Abstract

**Background:**

Metastatic pancreatic tumors from lung cancer (MPTLC) constitute 3% of all metastatic pancreatic tumors. We present an extremely rare case of cystic MPTLC that was difficult to distinguish from intraductal papillary mucinous neoplasm (IPMN).

**Case presentation:**

The patient was a 74-year-old woman who underwent lobectomy of lung cancer 2 years before presentation to our hospital. She was referred to our department for resection of cystic pancreatic tumors, which were diagnosed as IPMN with high-risk stigmata. Abdominal computed tomography (CT) showed a 37-mm-wide cystic tumor with a contrasted solid nodule in the pancreatic head and a 17-mm-wide cystic tumor in the pancreatic tail. We performed a total pancreatectomy for these lesions. According to histopathological and immunohistochemical findings, the tumors were diagnosed as metastatic pancreatic tumors from lung cancer.

**Conclusion:**

In this case, the cystic morphology was formed by eosinophilic secretions from tumor cells, and it was difficult to distinguish from IPMN with high-risk stigmata. We consider this case, based on the variable clinical findings, an extremely rare variant of MPTLC.

## Background

Metastatic pancreatic tumors from lung cancer (MPTLC) constitute 3% of all metastatic pancreatic tumors [1]. Although MPTLC is mainly treated with chemotherapy, pancreatectomy is sometimes performed in cases of solitary or metachronal metastasis. MPTLC is reported to present as hypovascular or ring-enhancing lesions on imaging findings [2], but it is difficult to distinguish from primary pancreatic cancer. Because MPTLC typically forms solid tumors, cystic changes of MPTLC are extremely rare. Herein, we reported a case of cystic MPTLC, which was difficult to distinguish from intraductal papillary mucinous neoplasm (IPMN).

## Case presentation

**Fig. 1:**
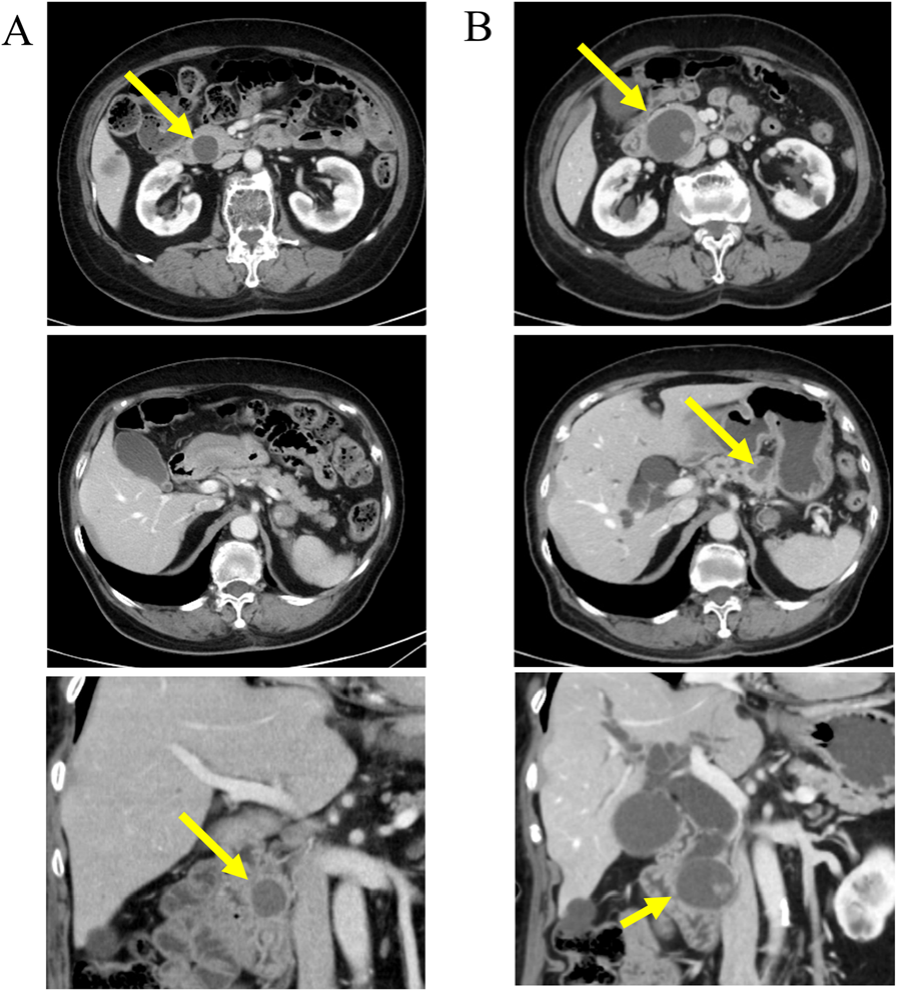
Enhanced computed tomography findings. **a** CT findings at 1 year after lobectomy for primary lung cancer. A 20-mm cystic tumor was detected on the pancreatic head, with no tumor on the pancreatic body. **b** CT findings at 2 year after lobectomy. The cystic tumor on the pancreatic head gradually increased from 20 mm to 37 mm and showed a contrasted solid nodule inside the cystic tumor. The cystic tumor was also indicated at the pancreatic body

**Fig. 2:**
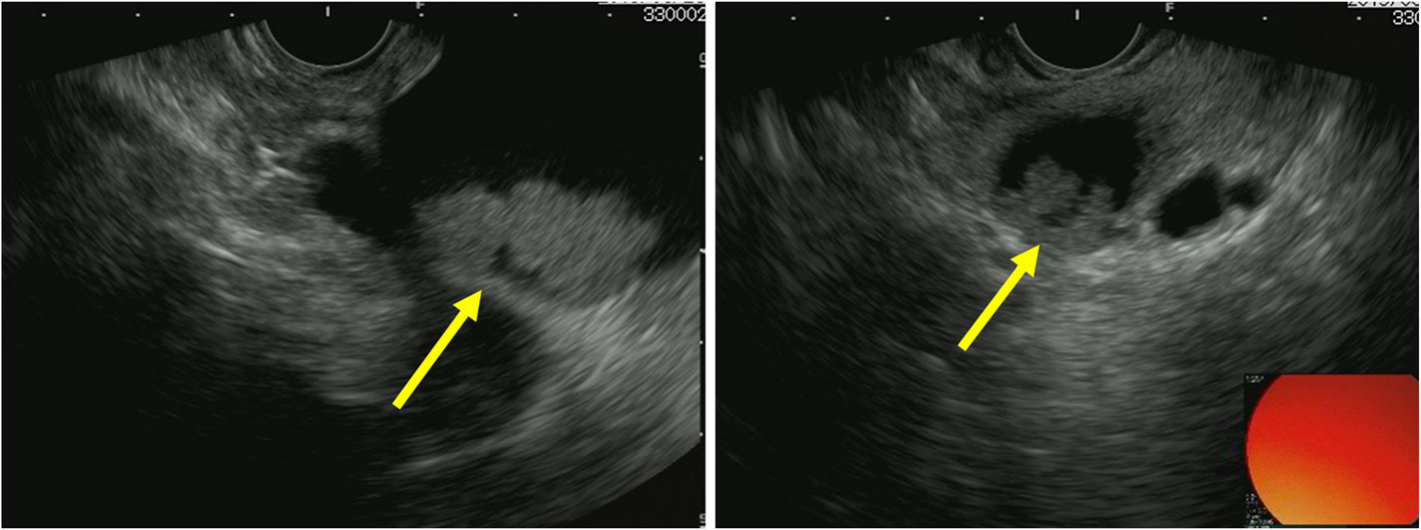
Endoscopic ultrasonography (EUS) imaging findings. EUS revealed that the cystic tumor of the pancreatic head was a 35-mm solitary cyst with a 24-mm mural nodule, and the cystic tumor of the pancreatic tail was a 20-mm solitary cyst with a mural nodule

**Fig. 3:**
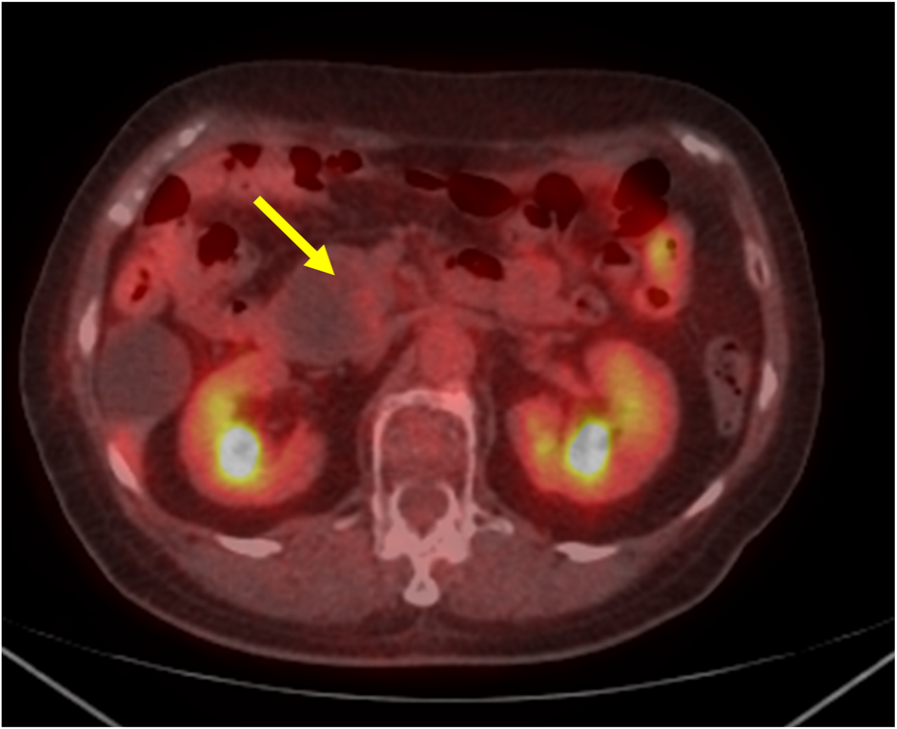
^18^F-Fluorodeoxyglucose positron-emission tomography findings (FDG-PET). FDG-PET showed FDG uptake (SUV max 1.9) in the lesion at the pancreatic head. No evidence of metastasis from other organs was observed

**Fig. 4:**
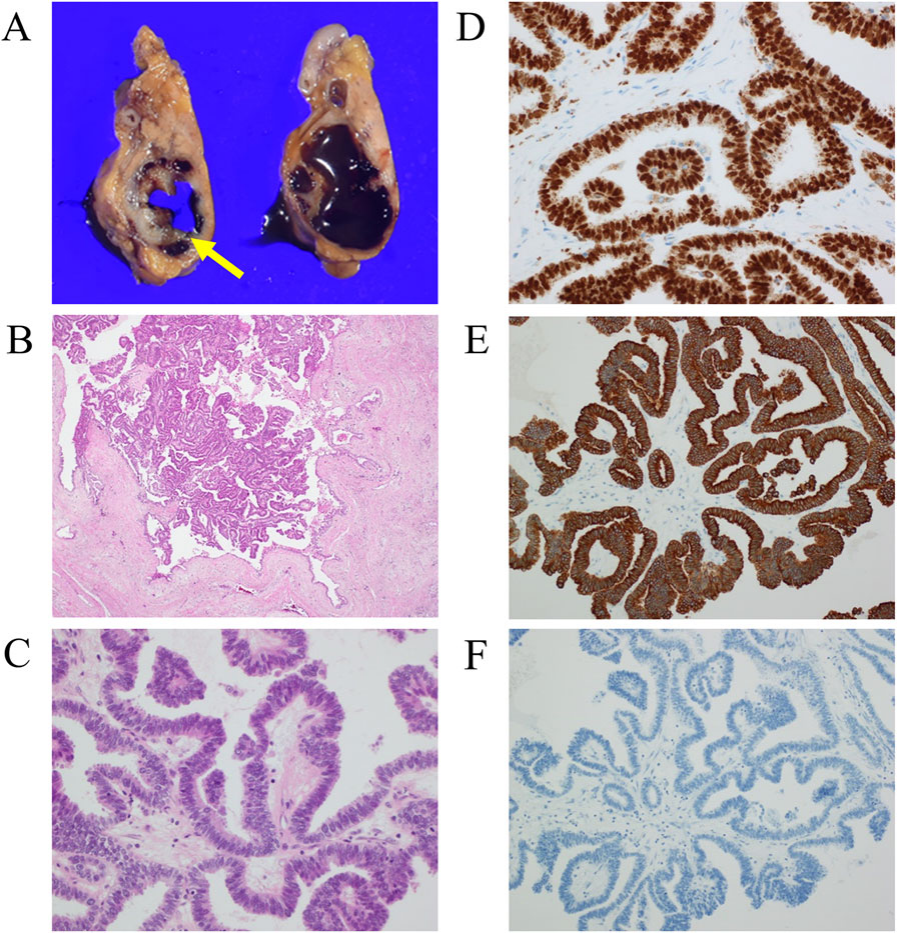
Histopathological findings of pancreatic tumors. **a** Macroscopic findings showed that the tumor comprised of a cystic wall and mural nodule, which contained cystic fluid. **b** Hematoxylin-eosin staining (× 40). Papillary tumors were growing in large cysts. Tumor cells had no mucus in the cytoplasm and had different characteristics from intraductal papillary mucinous neoplasm. The papillary structure was similar to that of the previously resected lung cancer tissue. **c** Hematoxylin-eosin staining (× 200) showed the power field of **a**. **d** Multiplex immunohistochemical staining (× 400), using specific antibody cocktails of TTF-1 and Napsin A. Nuclear expression of TTF-1 and granular cytoplasmic expression of Napsin A were seen. **e** CK7 was positive by immunohistochemical staining. **f** CK20 did not present significant staining

**Fig. 5:**
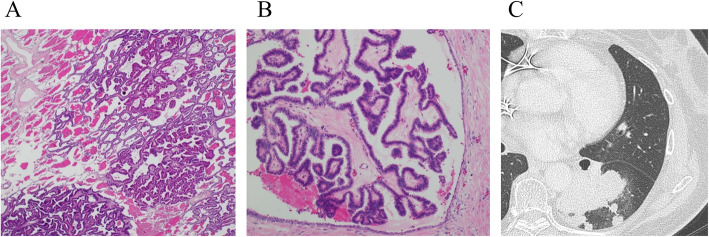
Histopathological and computed tomography (CT) findings of lung adenocarcinoma that was resected 2 years before pancreatectomy. **a** Hematoxylin-eosin staining (× 40). This figure shows a part, including the boundary with the non-tumor part. Tumors formed papillary structures in some places, and there were also alveolar epithelial replacement growths. **b** Hematoxylin-eosin staining (× 200) showed the power field of **a**. **c** Lung cancer had been detected at the left lower lobe as a solid tumor by CT

The patient was a 74-year-old female who underwent left lower lobectomy for lung cancer 2 years before presenting to our institution. The histological type was adenocarcinoma, with a pathological staging of T4N1M0 stage IIIA (Union for International Cancer Control: UICC 8th ed) [3]. One year after lobectomy, cystic lesions appeared on the head and tail of the pancreas, diagnosed as IPMN. The cystic tumor on the pancreatic head gradually increased from 20 to 37 mm in 1 year and showed a contrasted solid nodule inside the cystic tumor (Fig. 1). The patient was referred to our department for surgery because the tumor was considered IPMN with high-risk stigmata. Her blood test results were as follows: carcinoembryonic antigen, 2.4 ng/mL (normal range, < 5.0 ng/mL); carbohydrate antigen 19-9, 38 U/mL (normal range, < 15 U/mL); DUPAN-2, 39 U/mL (normal range, < 150 U/mL); and SPAN-1, 29.8 U/mL (normal range, < 30 U/mL). Abdominal computed tomography (CT) showed a 37-mm cystic tumor with a contrasted solid nodule at the pancreatic head and a 17-mm cystic tumor at the pancreatic tail. Endoscopic ultrasonography (EUS) revealed that the cystic tumor at the head was a 35-mm solitary cyst with a 24-mm mural nodule, and the cystic tumor at the tail was a 20-mm solitary cyst with a 10-mm mural nodule. The main pancreatic duct had no extension. Although we had confirmed that the cystic tumor and main pancreatic duct were close, we could not define the link between the main pancreatic duct and the cyst (Fig. 2). ^18^F-fluorodeoxyglucose positron-emission tomography (FDG-PET) showed FDG uptake (SUV max 1.9) at the lesion in the pancreatic head. No evidence of metastasis from other organs was observed (Fig. 3). Magnetic resonance imaging (MRI) could not be performed because of a cardiac pacemaker. The patient developed jaundice because the pancreatic head tumor excluded the common bile duct. From these results, we diagnosed the tumors as IPMN with high-risk stigmata because of jaundice and a contrasted mural nodule. We performed a total pancreatectomy for the two lesions after bile duct drainage by endoscopic retrograde cholangiopancreatography (ERCP). Because we performed ERCP on emergency, we could not perform brushing cytology or pancreatic juice cytology for food residue in the stomach and duodenum. The tumors were solitary cysts with papillary lesions at the pancreatic head and tail. Histopathological findings showed that tumor cells had papillary components without mucus production (Fig. 4a, b, c). Moreover, a small tumor lesion was also microscopically detected at the pancreatic tail. Immunohistochemical analysis showed positive results for TTF-1, Napsin A, and CK7, but CK20 did not present significant staining (Fig. 4d, e, f), and these findings indicated this tumor to be lung cancer metastasis rather than IPMN. The histological findings were similar to those of the existing lung adenocarcinoma resected 2 years before now (Fig. 5). According to these findings, we diagnosed the patient with metastatic pancreatic carcinoma from lung cancer. The postoperative course was good, and the patient was discharged 21 days after the operation. The patient did not received adjuvant therapy and had no recurrence for 6 months after pancreatectomy.

## Discussion

Metastatic pancreatic tumors are reported to consist of renal cell carcinoma, breast cancer or colorectal cancer, and lung cancer, which accounts for 3% of all cases [1]. On the other hand, lung cancer metastasizes to the pancreas at a frequency of 13.8%, and small cell carcinoma is the most frequent histological type [4].

In imaging diagnosis, metastatic pancreatic tumors are reported to reflect the features of primary lesions, and pancreatic metastases of renal cell carcinoma are relatively easy to diagnose as they are hypervascular tumors, like the primary tumor [5]. MPTLC is reported to have several features, such as excluding stenosis and semilunar disruption of the main pancreatic duct by endoscopic retrograde cholangiopancreatography or hypovascular tumors and ring-enhancing images by enhanced CT/MRI [2]. Rumancik et al. have argued that it is difficult to distinguish MPTLC from primary pancreatic cancer in diagnostic imaging [6]. Recently, several studies reported the feasibility of endoscopic ultrasound-guided fine-needle aspiration (EUS-FNA) to confirm diagnoses [7, 8]. Cystic MPTLC is extremely rare, with few reports in the literature. Ramirez et al. reported cystic MPTLC cases that showed metastasis 2 years after lobectomy for undifferentiated large-cell lung carcinoma [9].

IPMN is defined as a pancreatic tumor producing mucus with a papillary epithelial structure [10]. IPMN is classified as having worrisome features or high-risk stigmata from several malignant risk factors [11]. The 2017 revision of the International Association of Pancreatology consensus guidelines suggested that high-risk stigmata were obstructive jaundice, an enhancing mural nodule ≥ 5 mm, and dilation of the main pancreatic duct to a diameter of ≥ 10 mm. Because our case had features of enlargement, obstructive jaundice, and papillary nodules inside cystic lesions, it was difficult to distinguish MPTLC from IPMN with high-risk stigmata. Moreover, EUS-FNA was difficult to perform in this case due to cystic changes. In this patient, the case findings suggested a chronologically increasing cyst with the appearance of an enhancing mural nodule, and these were the basis for considering it IPMN with high-risk stigmata. On the other hand, it was untypical of IPMN that dilation of the main pancreatic duct was not observed with the increase in cyst size.

Reddy and Wolfgang proposed the following surgical indications for metastatic pancreatic tumors: (i) a relatively better prognosis of the primary lesion, (ii) a controlled primary lesion, (iii) absence of multiple metastases, (iv) a resectable metastatic lesion, and (v) an operable condition of the patient [1]. A standard pancreatectomy with lymph node dissection is recommended to prevent recurrence. Although the resection of MPTLC was not recommended because of poor improvements in prognosis [12], Dietzek et al. reported good surgical indications for MPTLC with long intervals between initial therapy and recurrence [13]. Masetti et al. evaluated the prognoses of 234 pancreatic metastasis cases, and poor prognostic factors included being symptomatic, having multiple metastases, and incomplete resection in univariate analysis, and incomplete resection and melanoma in multivariate analysis [14]. Pancreatic metastasis of renal cell carcinoma had a significantly better prognosis than other cancers. MPTLCs have been treated with surgical resection in cases of metastases from adenocarcinoma or squamous cell carcinoma, but metastases from small cell carcinoma were mostly treated with chemotherapy.

In this case, adenocarcinoma was diagnosed as the primary lesion, which showed eosinophilic secretions pathologically. In the pancreatic lesions, no mucus was found in the cyst components or tumor cells, suggesting the accumulation of eosinophilic secretions in the tumor as in the primary lesion. These eosinophilic secretions and papillary nodules in the cyst exhibited a morphology that resembled that of IPMN with high-risk stigmata. Regarding the diagnosis, the histological and immunohistochemical similarities (TTF-1, Napsin A, and CK7 were positive) with lung cancer were comprehensively evaluated, and this tumor could be diagnosed as an MPTLC. Several pathways have been reported for metastatic pancreatic tumors, including direct invasion from surrounding organs, lymphatic metastasis to the peri-pancreatic lymph node, or hematogenous metastasis [15]. Because this case had no metastasis in 53 pieces of dissected lymph node, hematogenous metastasis is most likely the implicated pathway. As a characteristic clinical course of this case, the progression rate was rapid for IPMN. This case was labeled as metachronous metastasis, and radical resection was performed due to multiple lesions in the pancreas. Therefore, it is necessary to monitor for recurrence in the future carefully.

## Conclusion

In this case, the cystic morphology was formed by eosinophilic secretions from tumor cells, and it was difficult to distinguish from IPMN with high-risk stigmata. We consider this case, based on the variable clinical findings, an extremely rare variant of MPTLC.

## Data Availability

The dataset supporting the conclusions of this article is included in the article.

## Abbreviations

MPTLC: Metastatic pancreatic tumor from lung cancer
IPMN: Intraductal papillary mucinous neoplasm
EUS-FNA: Endoscopic ultrasound-guided fine needle aspiration
FDG-PET: ^18^F-Fluorodeoxyglucose positron-emission tomography
CT: Computed tomography
MRI: Magnetic resonance imaging
